# Analysis of environmental risk factors for chronic obstructive pulmonary disease exacerbation: A case-crossover study (2004-2013)

**DOI:** 10.1371/journal.pone.0217143

**Published:** 2019-05-23

**Authors:** Javier de Miguel-Díez, Julio Hernández-Vázquez, Ana López-de-Andrés, Alejandro Álvaro-Meca, Valentín Hernández-Barrera, Rodrigo Jiménez-García

**Affiliations:** 1 Pneumology Department, Hospital General Universitario Gregorio Marañón, Universidad Complutense de Madrid, Madrid, Spain; 2 Pneumology Department, Hospital Universitario Infanta Leonor, Madrid, Spain; 3 Preventive Medicine and Public Health Teaching and Research Unit, Department of Health Sciences, Universidad Rey Juan Carlos, Alcorcón, Madrid, Spain; Telethon Institute for Child Health Research, AUSTRALIA

## Abstract

**Purpose:**

We aim to assess if air pollution levels and climatological factors are associated with hospital admissions for exacerbation of chronic obstructive pulmonary disease (COPD) in Spain from 2004 to 2013.

**Methods:**

We conducted a retrospective study. Information on pollution level and climatological factors were obtained from the Spanish Meteorological Agency and hospitalizations from the Spanish hospital discharge database. A case-crossover design was used to identify factors associated with hospitalizations and in hospital mortality. Postal codes were used to assign climatic and pollutant factors to each patient.

**Results:**

We detected 162,338 hospital admissions for COPD exacerbation. When seasonal effects were evaluated we observed that hospital admissions and mortality were more frequent in autumn and winter. In addition, we found significant associations of temperature, humidity, ozone (O_3_), carbon monoxide (CO), particulate matter up to 10 μm in size (PM_10_) and nitrogen dioxide (NO_2_) with hospital admissions. Lower temperatures at admission with COPD exacerbation versus 1, 1.5, 2 and 3 weeks prior to hospital admission for COPD exacerbation, were associated with a higher probability of dying in the hospital. Other environmental factors that were related to in-hospital mortality were NO_2_, O_3_, PM_10_ and CO.

**Conclusions:**

Epidemiology of hospital admissions by COPD exacerbation was negatively affected by colder climatological factors (seasonality and absolute temperature) and short-term exposure to major air pollution (NO_2_, O_3_, CO and PM_10_).

## Introduction

Chronic obstructive pulmonary disease (COPD) is a major cause of health care costs, mortality and morbidity around the world. Exacerbations of existing COPD are a frequent reason for hospital admission and with increased mortality among these patients [1]. Known risk factors for COPD exacerbations include exposures to tobacco, some workplace exposures, and infections by bacteria and virus [2]. Another potential trigger for such exacerbations is short-term exposures to air pollution [3–5].

The evidence regarding the effects of air pollution exposure on COPD exacerbations is still limited. It has been investigated by several studies. Some of them have reported that air pollutions are risk factor for exacerbation or mortality [6–8], while others found not significant associations or relationship only for selected populations [9,10]. However, a recent systematic review and meta-analysis concluded that the risk of COPD exacerbations is significantly increased by short-term exposure to major air pollutants [11].

The mechanisms by which COPD exacerbations can be triggered after exposure to air pollution are not yet fully understood. However, there are several reasonable hypotheses [12]. Particulate matters like particulate matter up to 10 μm in size (PM_10_) and gaseous pollutants of nitrogen dioxide (NO_2_) and ozone (O_3_) can all produce deleterious effects on the respiratory airways such as increased bronchial reactivity [13], airway oxidative stress-induced DNA damage [14,15], pulmonary and systemic inflammation [16–18], amplification of viral infections [19], and reduction in airway cialiary activity [20]. On the other hand, sulfur dioxide (SO_2_) is a well-known respiratory irritant, with acute respiratory symptoms reported immediately upon exposure to elevated concentrations, and it can also cause bronchoconstriction [2,21].

Extremes of temperature, both cold and heat, have also been associated to excess mortality and morbidity among patients suffering COPD [22]. However, the interaction between temperature and air pollution among COPD patients and the effect such interaction has on the morbidity burden has been poorly investigated so far [23].

We aim to assess if air pollution levels and climatological factors are associated with hospital admissions for exacerbation of chronic obstructive pulmonary disease (COPD) in Spain from 2004 to 2013.

## Material and methods

### Study population

We conducted a retrospective study. All patients hospitalized in Spain from 1 January 2004 to 31 December 2013 for suffering a COPD exacerbation were included in our investigation. Hospital admissions were extracted from the Spanish National Hospital Discharge data base (Spanish Minimum Basic Data Set, MBDS). Over 97% of all hospitalizations in Spain are included in this database. Details on the MBDS can be found elsewhere [24].

The MBDS variables used for our investigation included; sex, age, dates of hospital admission date of hospital discharge, postal code of the patient, up to 14 diagnosis and 20 procedure codes and outcome at discharge. The *9*^*th*^ *edition of the International Classification of Diseases* (ICD-9-CM) is used for coding in the MBDS. We excluded patients with a missing postal code.

### Environmental data

Information on pollution level and climatological factors were obtained from the Spanish Meteorological Agency (AEMET) (http://www.aemet.es/). As we did not have any individual exposure levels postal codes were used to assign climatic and pollutant factors to each patient using the data provided from the nearest station to the patient’s residence.

The daily data assigned to each patient included, humidity, temperature and the following pollutants PM_10_, SO_2_, NO_2_, O_3_, and CO, There are around 800 meteorological stations in Spain. The locations of these meteorological stations for each of the 17 Spanish Autonomous Communities can be obtained from the web of the AEMET (http://www.aemet.es/es/eltiempo/observacion).

### Outcome variables

We considered the main outcome variable a hospital admission with a primary diagnosis of COPD exacerbation (code 491.21 in the ICD9CM). According to the MBDS methodology for every patient admitted to the hospital, and beside which is the primary diagnosis, those who have been admitted to any hospital in the previous 30 days are considered a readmission. In our study all patients who had an admission in the previous month were deleted from the database so it is not possible to have an overlap with other admission in which COPD was not the primary diagnosis.

### Statistical analysis

The statistical methods used have been described in detail in a previous study conducted by our group. [25] Basically the following processes were conducted. To assess the seasonal effect on COPD exacerbation that resulted in a hospital admission the years were divided into quarters. We used a Bayesian model with Poisson distribution to analyze the seasonal effect [26].

To evaluate the effect of each environmental factor on the hospital admissions for COPD exacerbation we used a case-crossover design (CCD) [25, 27, 28] In this design each patient is used as his own control. We considered four time periods before the date of the hospital admission (baseline) for each patient as control periods (1, 1.5, 2 and 3 weeks) as has been described before [29]. In order to avoid the effect of one day with outlier values we calculated the mean for each environmental factor including the day before and after the control periods and for the baseline the two days immediately before. The association between environmental factors and COPD exacerbation admissions was evaluated using conditional logistic regression obtaining odds ratios with their 95%CI using an exact method. To construct each model we introduced the environmental factor under study and afterwards introduced the remaining factors one by one in the model for adjustment. If any of the remaining factor had a significant correlation with the environmental factor under study this factor was excluded from the model.

R statistical package version 3.4.4 (GNU General Public License) was used for all analyses and a p-values <0.05 (two-tailed) was the cut point for significance [30]

### Ethical aspects

The study maintains data confidentiality at all times. Given the anonymous and mandatory nature of the database, it was not necessary to obtain informed consent or approval by an ethics committee in accordance with Spanish legislation.

## Results

### Characteristics of study population

The epidemiological and clinical characteristics of the study population are shown in Table 1. We detected 162,338 hospital admissions for COPD exacerbation. The median age was 75.1 years and 83.5% were male. The most frequent comorbidities were mild diabetes without complication (24.5%), congestive heart failure (19.3%), and renal disease (11.1%).

**Table 1:** Epidemiological and clinical characteristics of patients admitted to hospital with a COPD exacerbation in Spain from 2004 to 2013.

Description	Data
No. of patients	162338
Males	135598 (83.5)
Age (years)	75.15 (10.76)
Length of stay (days)	8.37 (7.73)
Charlson index	2.42 (1.73)
In-hospital mortality	9868 (6.1)
**Comorbid diseases**	
Myocardial infarction	6404 (3.9)
Congestive heart failure	31275 (19.3)
Peripheral vascular disease	9087 (5.6)
Cerebrovascular disease	6680 (4.1)
Dementia	4226 (2.6)
Connective tissue disease-rheumatic disease	2394 (1.5)
Peptic ulcer disease	1177 (0.7)
Mild liver disease	7463 (4.6)
Diabetes without complications	39749 (24.5)
Diabetes with complications	2843 (1.8)
Paraplegia and hemiplegia	453 (0.3)
Renal disease	18063 (11.1)
Cancer	10876 (6.7)
Moderate or severe liver disease	760 (0.5)
Metastatic carcinoma	2593 (1.6)

Values are expressed as absolute number (percentage) and mean (95% of confidence interval).

### Effect of season on admission with COPD exacerbation and in-hospital mortality

**Fig 1:**
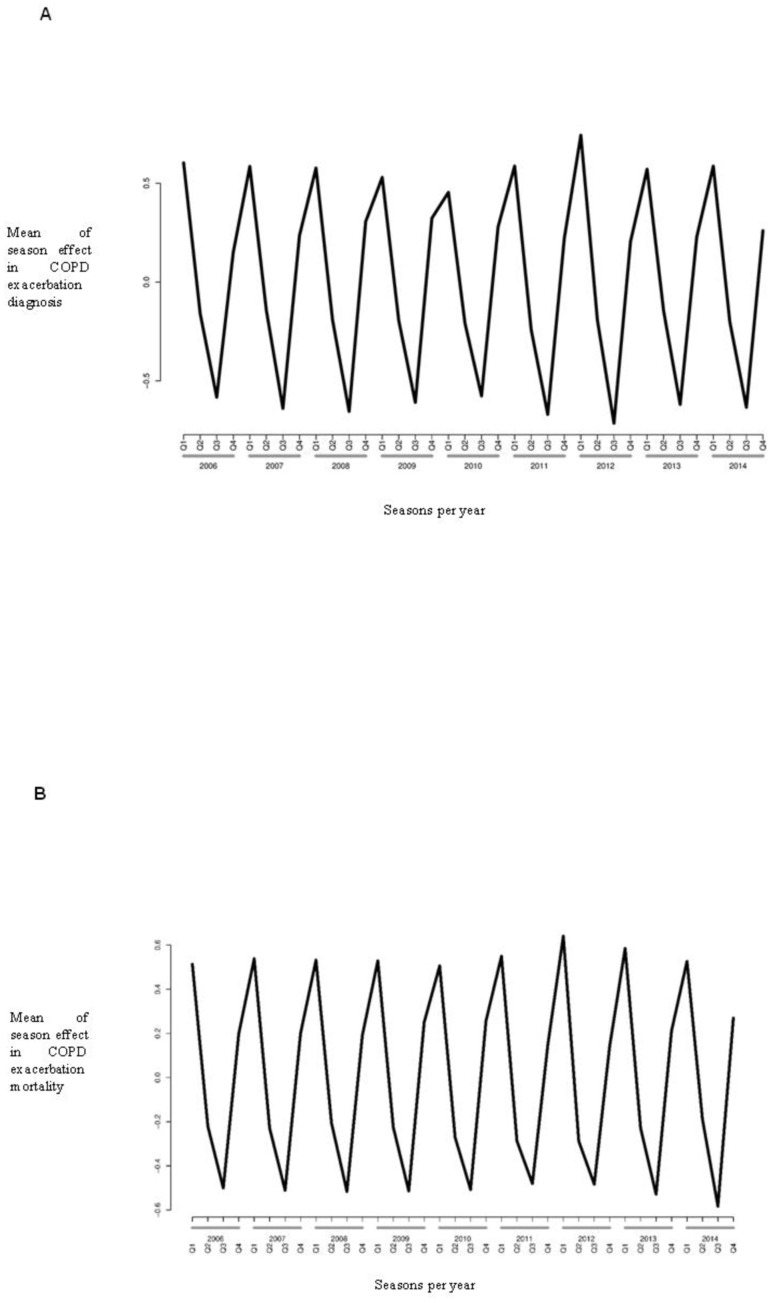
Mean of the seasonal effect in admissions for COPD exacerbation (A) and in-hospital mortality (B) in Spain between 2004 and 2013.

When seasonal effects were evaluated using a Bayesian model (Fig 1A), COPD exacerbation admissions were less common in second and third quarter of the year, and more abundant in the last and first quarter of the year. Similar pattern were found in COPD exacerbation -related death (Fig 1B).

### Effects of short-term exposure to environmental risk factors on COPD exacerbation hospital admissions and in-hospital mortality

In the bivariate model, significant associations were found for temperature, NO_2_, O_3_, PM_10_ and CO with COPD exacerbation hospital admissions. Using a multi-environmental factor model temperature, humidity, NO_2_, O_3_, PM_10_ and CO remained significantly associated (Table 2).

**Table 2:** Bivarite and multivariable models results for the associations between environmental factors and COPD exacerbation hospital admissions for study time periods (1, 1.5, 2 and 3 weeks before hospitalization).

	Unadjusted		Adjusted	
Environmental factor (Unit)	OR (95% CI)	p-value	OR (95% CI)	p-value
**1 week**				
Temperature (°C)	0.99 (0.99; 0.99)	**<0.001**	0.99 (0.98; 0.99)	**<0.001**
Humidity (%)	0.99 (0.99; 1.00)	0.117	0.99 (0.98; 0.99)	**0.002**
NO2 (Bg/m3)	1.02 (1.01; 1.03)	**0.020**	1.01 (0.99; 1.03)	0.229
SO2 (Bg/m3)	1.00 (0.99; 1.01)	0.641	1.00 (0.99; 1.01)	0.711
O3 (Bg/m3)	0.98 (0.98; 0.99)	**<0.001**	0.98 (0.97; 0.99)	**<0.001**
PM10 (Bg/m3)	0.99 (0.98; 1.01)	0.511	0.98 (0.97; 0.99)	0.034
CO (Bg/m3)	1.04 (1.03; 1.05)	**<0.001**	1.04 (1.02; 1.06)	**<0.001**
**1.5 weeks**				
Temperature (°C)	0.99 (0.99; 1.01)	0.167	0.99 (0.99; 0.99)	**0.024**
Humidity (%)	1.00 (0.99; 1.01)	0.475	0.99 (0.99; 1.01)	0.236
NO2 (Bg/m3)	0.97 (0.96; 0.98)	**<0.001**	0.97 (0.96; 0.98)	**<0.001**
SO2 (Bg/m3)	0.99 (0.99; 1.01)	0.503	1.00 (0.99; 1.01)	0.908
O3 (Bg/m3)	0.99 (0.98; 0.99)	**<0.001**	0.98 (0.97; 0.99)	**<0.001**
PM10 (Bg/m3)	0.96 (0.95; 0.97)	**<0.001**	0.96 (0.94; 0.97)	**<0.001**
CO (Bg/m3)	1.03 (1.02; 1.04)	**<0.001**	1.04 (1.03; 1.06)	**<0.001**
**2 weeks**				
Temperature (°C)	0.99 (0.99; 0.99)	**<0.001**	0.99 (0.99; 0.99)	**<0.001**
Humidity (%)	0.99 (0.99; 1.01)	0.440	0.99 (0.99; 0.99)	**0.043**
NO2 (Bg/m3)	1.03 (1.01; 1.04)	**<0.001**	1.02 (1.01; 1.04)	**0.002**
SO2 (Bg/m3)	0.99 (0.99; 1.01)	0.187	0.99 (0.99; 1.01)	0.107
O3 (Bg/m3)	0.98 (0.97; 0.98)	**<0.001**	0.98 (0.97; 0.99)	**<0.001**
PM10 (Bg/m3)	0.99 (0.98; 1.01)	0.222	0.97 (0.96; 0.99)	**0.001**
CO (Bg/m3)	1.05 (1.03; 1.06)	**<0.001**	1.04 (1.03; 1.06)	**<0.001**
**3 weeks**				
Temperature (°C)	1.01 (1.01; 1.01)	**0.029**	1.00 (0.99; 1.00)	0.206
Humidity (%)	1.00 (1.00; 1.00)	0.858	1.00 (1.00; 1.00)	0.650
NO2 (Bg/m3)	1.03 (1.02; 1.05)	**<0.001**	1.02 (1.01; 1.03)	**0.017**
SO2 (Bg/m3)	0.99 (0.99; 1.00)	0.715	0.99 (0.99; 1.01)	0.551
O3 (Bg/m3)	0.97 (0.97; 0.98)	**<0.001**	0.97 (0.97; 0.98)	**<0.001**
PM10 (Bg/m3)	1.01 (0.99; 1.02)	0.452	0.98 (0.97; 0.99)	**0.026**
CO (Bg/m3)	1.05 (1.04; 1.06)	**<0.001**	1.05 (1.03; 1.06)	**<0.001**

NO_2_, nitrogen dioxide; SO_2_, sulfur dioxide; O_3_, ozone; PM_10_, particulate matter up to 10 μg/ m^3^ in size; CO: carbon monoxide; OR, odds ratio; 95% CI, 95% of confidence interval.

Both CO and NO_2_ concentrations showed, in general, significant OR values >1. Thus, high concentrations of CO and NO_2_ at the time of admission when they were taken as control 1.5, 2 and 3 weeks before admission were significantly associated with a higher possibility of COPD exacerbation hospital admission. Concentrations of O_3_, PM_10_ showed values of OR <1. Specifically, high concentrations of O_3_ and PM_10_ 1, 1.5, 2 and 3 weeks before admissions were significantly associated with high probability of COPD exacerbation related hospitalization. Note that associations of humidity and temperature with COPD exacerbation hospitalization only occurred when 1 and 2 weeks were considered as control times; thus, low temperatures at the time of admission, with respect to the corresponding control times, were significantly associated with a greater possibility of hospital admission related to COPD exacerbation. SO_2_ concentrations were not associated with COPD exacerbation hospital admissions in any of the periods analyzed.

The effects of each environmental factor in the in-hospital mortality after COPD exacerbation are shown in Table 3.

**Table 3:** Bivarite and multivariable models results for the associations between environmental factors and in hospital mortality after COPD exacerbation hospital admissions for study time periods (1, 1.5, 2 and 3 weeks before hospitalization).

	Unadjusted		Adjusted	
Environmental factor (Unit)	OR (95% CI)	p-value	OR (95% CI)	p-value
**1 week**				
Temperature (°C)	0.98 (0.97; 0.99)	**<0.001**	0.98 (0.97; 0.99)	**<0.001**
Humidity (%)	0.99 (0.99; 1.01)	0.526	1.00 (0.99; 1.00)	0.096
NO2 (Bg/m3)	0.98 (0.93; 1.04)	0.567	0.98 (0.92; 1.04)	0.475
SO2 (Bg/m3)	0.99 (0.97; 1.02)	0.681	1.01 (0.98; 1.04)	0.516
O3 (Bg/m3)	0.96 (0.93; 0.99)	**0.011**	0.95 (0.92; 0.99)	**0.015**
PM10 (Bg/m3)	0.95 (0.90; 1.01)	0.112	0.94 (0.88; 1.00)	**0.051**
CO (Bg/m3)	1.05 (0.99; 1.12)	0.062	1.05 (0.99; 1.12)	0.099
**1.5 weeks**				
Temperature (°C)	0.98 (0.98; 0.99)	**<0.001**	0.98 (0.97; 0.99)	**<0.001**
Humidity (%)	1.00 (1.00; 1.00)	0.349	1.00 (1.00; 1.00)	0.959
NO2 (Bg/m3)	0.91 (0.87; 0.96)	**<0.001**	0.92 (0.87; 0.98)	**0.007**
SO2 (Bg/m3)	0.98 (0.96; 1.01)	0.183	1.00 (0.97; 1.02)	0.826
O3 (Bg/m3)	0.97 (0.94; 0.99)	**0.029**	0.95 (0.91; 0.98)	**0.002**
PM10 (Bg/m3)	0.89 (0.84; 0.94)	**<0.001**	0.89 (0.83; 0.95)	**<0.001**
CO (Bg/m3)	1.03 (0.98; 1.08)	0.226	1.04 (0.99; 1.09)	0.139
**2 weeks**				
Temperature (°C)	0.99 (0.98; 0.99)	**0.003**	0.99 (0.98; 0.99)	**<0.001**
Humidity (%)	1.00 (0.99; 1.01)	0.886	0.99 (0.99; 1.01)	0.473
NO2 (Bg/m3)	0.99 (0.94; 1.04)	0.626	0.99 (0.93; 1.05)	0.704
SO2 (Bg/m3)	0.99 (0.96; 1.01)	0.289	0.99 (0.96; 1.02)	0.427
O3 (Bg/m3)	0.96 (0.93; 0.99)	**0.006**	0.96 (0.93; 0.99)	**0.018**
PM10 (Bg/m3)	0.95 (0.89; 1.01)	0.065	0.94 (0.88; 1.00)	**0.053**
CO (Bg/m3)	1.03 (0.98; 1.08)	0.208	1.04 (0.98; 1.09)	0.198
**3 weeks**				
Temperature (°C)	0.99 (0.98; 0.99)	**0.012**	0.99 (0.98; 0.99)	**0.004**
Humidity (%)	1.00 (0.99; 1.00)	0.767	0.99 (0.99; 1.00)	0.704
NO2 (Bg/m3)	1.03 (0.98; 1.08)	0.293	1.00 (0.94; 1.06)	0.946
SO2 (Bg/m3)	0.99 (0.97; 1.01)	0.436	0.99 (0.97; 1.02)	0.713
O3 (Bg/m3)	0.95 (0.92; 0.97)	**<0.001**	0.94 (0.91; 0.98)	**0.001**
PM10 (Bg/m3)	0.97 (0.92; 1.03)	0.306	0.94 (0.88; 1.00)	0.058
CO (Bg/m3)	1.06 (1.01; 1.11)	**0.011**	1.06 (1.01; 1.12)	**0.035**

NO_2_, nitrogen dioxide; SO_2_, sulfur dioxide; O_3_, ozone; PM_10_, particulate matter up to 10 μg/ m^3^ in size; CO: carbon monoxide; OR, odds ratio; 95% CI, 95% of confidence interval.

Lower temperatures at admission with COPD exacerbation versus 1, 1.5, 2 and 3 weeks before hospital admission for COPD exacerbation, increased the risk of in-hospital mortality in both crude and adjusted analysis. Other environmental factors that were related to in-hospital mortality were NO_2_, O_3_, PM_10_ and CO. No significant association between SO_2_ concentrations and in-hospital mortality in patients admitted for COPD exacerbation were found.

## Discussion

Our study provides evidence that the epidemiology of hospital admissions by COPD is associated with environmental factors. We observed an overall seasonal effect as most hospital admissions for COPD exacerbation and in-hospital mortality occurred in the colder seasons (autumn to winter). Moreover, lower temperatures and higher concentrations of atmospheric pollutants significantly increased the incidence of hospitalization and in-hospital mortality.

We also described for all the time periods analyzed a significant association between short-term exposure to concentrations of NO_2_, O_3_ and PM10 and hospital admission for COPD exacerbation. Studies conducted in Europe and US agree with us observing the association between hospital admissions for COPD exacerbation and air pollution [31–36]. Additionally in a recent systematic review, Devries et al [2] reported that the incidence of hospitalization and of emergency room visits related to COPD showed a significant increase as the concentration of PM_2.5_, NO_2_, and SO_2_ raised. Another recent meta-analysis confirmed found that short-term exposure to gaseous and particulate pollutants increased the risk of COPD exacerbations significantly, 1% for PM_2.5_, 2% for NO_2_, and 0% for O_3_ for an increment of 10 μg/m^3^, but 0% for CO per increment of 100 μg/m [3,11]. Moreover, subgroup analysis according to age, study design, location and outcome, obtained similar and significant results.

Exposure to SO_2_ did not show a significant association with hospital admission for COPD exacerbation in the present study. These findings are consistent with a previous study, in which no effect was identified for SO_2_ [36]. By contrast, Santus et al [37] found that SO_2_ increases emergency room admissions for COPD exacerbations. In any case, over the last years the concentrations of SO_2_ have decreased sharply, as a consequence of cleaner motor vehicles fuels so nowadays NO_2_, O_3_ and PM have become the more relevant pollutants from a heath point of view [15].

The current analysis also has found a significant association between higher probability of hospital admissions with COPD exacerbation and lower temperatures at admission. Similar results have been reported by Krachunov et al [38], who reported that the levels of air pollutants were associated with the lower daily mean temperatures. In this way, Almagro et al [39] reported a 4.7% increase in the incidence of hospitalizations per 1 degree decrease in the temperature. Thus, Aga et al [40] observed that the daily number of deaths increased by 0.8%. per 10 μg/m^3^ increase in PM_10_. However, the highest risk of mortality among COPD patients is not related to gas pollutants such as NO_2_ or O_3_, being particulate matter pollution the strongest factor [41].

Regarding in hospital mortality, we found the highest figures in cold seasons such as autumn and winter. It has been previously reported that deaths from COPD increase significantly during the cold weather found in winter [42], but this is usually considered as a cofactor in the investigation on the effect of atmospheric pollution [43,44]. Agreeing with this conclusion we found that, not only lower temperatures, but also higher NO_2_, O_3_, CO and PM_10_ concentrations in the weeks before the hospital admission for COPD exacerbation, increased the risk of in-hospital mortality.

Nevertheless, several limitations of this study must be considered. First, we did not measure exposure to other environmental agents such as PM_2.5_, which could also have an influence on hospital admissions for COPD exacerbations. The reason for this is that in Spain the number of stations measuring PM2.5 is much smaller than those measuring PM10, so we have used the latter for our study. Second, we measured exposure levels at the monitoring stations but not at the individuals’ home since we used zip codes not addresses. This is important as some gaseous pollutants from traffic such as NO2 can taper quickly from its source and the patient’s home distance from the monitoring stations is not known. Third, we have not included infections by influenza in Spain in our investigation, and is well known that influenza incidence is associated to temperature and humidity [45]. However, in Spain data on influenza is not available by postal code or even by province and the information is collected only from epidemiological week 40 to week 10 (22 out of 52 weeks). Fifth, in our study population we have a male predominance among COPD patient which is mainly due to secular effects of the tobacco exposure and has been described in previous Spanish investigations [46]. Sixth, we cannot use lag days and test which exposure has the strongest association with the outcome (day 0, day -1, day -2, etc) because as commented in the methods section we decided to use an average value for each environmental factor over a 3-day period. Finally, we don’t have certainty that those hospitalized with COPD exacerbation as their main diagnosis died in the hospital as a consequence of this disease.

In summary, our results showed that epidemiology of hospital admissions by COPD exacerbations was negatively affected by colder climatological factors (absolute temperature, and seasonality) and by the short-term exposure to major air pollution (NO_2_, O_3_, CO and PM_10_).

## Abbreviations

AEMET: Spanish State Meteorological Agency
CCD: case-crossover design
CLR: conditional logistic regression
CO: carbon monoxide
COPD: chronic obstructive pulmonary disease
ICD-9-CM: International Classification of Diseases, 9th ed, Clinical Modification
MBDS: Spanish Minimum Basic Data Set
MSSSI: Ministry of Health Social Services and Equality
NHS: National Health System
NO_2_: nitrogen dioxide
O_3_: ozone
PM_10_: particulate matter up to 10 μm in size
